# Prevalence and associated factors of soil-transmitted helminth infections among children in previous leprosarium and non-leprosarium areas in Eastern Ethiopia: A community-based comparative study

**DOI:** 10.1016/j.ijregi.2025.100633

**Published:** 2025-03-20

**Authors:** Fitsum Weldegebreal, Junedin Abamecha, Ukash Umer, Getachew Kabew Mekonnen, Assefa Desalew, Temam Beshir Raru, Kedir Urgesa

**Affiliations:** a School of Medical Laboratory Sciences, College of Health and Medical Sciences, Haramaya University, Harar, Ethiopia; b Laboratory Bacteriology Research, Department of Diagnostic Sciences, Faculty of Medicine and Health Sciences, Ghent University, Ghent, Belgium; c Department of Pediatrics and Child Health Nursing, School of Nursing, College of Health and Medical Sciences, Haramaya University, Harar, Ethiopia; d Department of Obstetrics and Gynecology, Leiden University Medical Centre, Leiden University, Leiden, The Netherlands; e School of Public Health, College of Health and Medical Sciences, Haramaya University, Harar, Ethiopia; f Deakin University, the Institute for Mental and Physical Health and Clinical Translation (IMPACT), School of Medicine, Barwon Health, Geelong, Victoria, Australia

**Keywords:** NTDs, STHs, Leprosy, Children, Ethiopia

## Abstract

**Objectives:**

Soil-transmitted helminths (STHs) infection is one of the neglected tropical diseases (NTDs) that mainly affect peoples living in resource-limited settings and poor sanitation. However, the extent and potential predisposing factors of this infection among peoples living in previous leprosarium settlements in Eastern Ethiopia are not well understood. Thus, the aim of this study was to compare the prevalence and associated factors of STHs infections among children aged 1-15 years in previous leprosarium and non-leprosarium areas in Eastern Ethiopia.

**Methods:**

A community-based comparative cross-sectional study was conducted among 580 systematically selected households from November 1, 2023 to February 30, 2024. A structured questionnaire was used to collect data on socio-demography, and associated factors of STHs infections. Approximately 5 g of stool sample was collected and processed using the formol-ether concentration technique. Data were entered in Epi-Data version 4.2, and analysis was performed using statistical package for social sciences (SPSS) version 26. A chi-square test was used to show statistical differences between the two groups. Binary logistic regression was performed to assess the association between independent variables and STHs infection.

**Results:**

The overall prevalence of STHs infection was 4.5% (95% confidence interval [CI] 1.31-16.80). The prevalence was 5.7% and 3.3% among children in previous leprosarium and non-leprosarium settlements, respectively (X^2^ = 7.98, *P* = 0.017). Having no habit of hand washing after helping and cleaning children who had defecated (adjusted odds ratio [AOR] = 1.26, 95% CI 1.20-5.80), (AOR = 4.90, 95% CI 2.21-9.33) and before eating (AOR = 3.25, 95% CI 1.40-11.23), (AOR = 18.70, 95% CI 9.21-38.21) was associated with STHs infection in both previous leprosarium and non-leprosarium settlements. However, having no habit of hand washing after toilet use (AOR = 1.80, 95% CI 1.34-7.40) and contact with soil (AOR = 5.98, 95% CI 1.62-22.10) was significantly associated with STHs infection in previous leprosarium settlements only.

**Conclusions:**

In this study, STHs infections disproportionately affect children living in previous leprosarium compared with non-leprosarium settlements. Therefore, targeted mass deworming and health information dissemination on proper hygiene and sanitation should be given to the community to alleviate the problem.

## Highlights

• The overall prevalence of soil-transmitted helminths (STHs) was 4.5%.
• The prevalence of STHs was 5.7% and 3.3% among children in former leprosy and non-leprosy settlements, respectively.
• Lack of hand washing after helping and cleaning children who had defecated and before eating was associated with STHs in both settlements.
• However, lack of hand washing after toilet use and contact with soil were associated with STHs only in former leprosy settlements.

## Introduction

Soil-transmitted helminths (STHs) infection remains a major public health problem in tropical countries despite it being classified as one of the neglected tropical diseases (NTDs) associated with poverty [1]. Parasitic co-infection and limited water, sanitation, and hygiene (WASH) have been suggested to predispose individuals to leprosy [2]. Available evidence suggests that helminthiasis may influence the transmission of leprosy in co-endemic areas. More than 94% of new annual leprosy cases are diagnosed in populations co-endemic for STH infections [3]. The STHs comprise *Ascaris lumbricoides, Trichuris trichiura*, and *Necator americanus*/*Ancylostoma duodenale* [4].

Globally, over two billion individuals are affected by STHs infections [5]. The most prevalent STH is *A. lumbricoides*, which affects approximately 1.2 billion individuals followed by *T. trichiura* and hookworm, affecting 795 and 740 million people, respectively [6]. In developing countries, infections with STHs are highly prevalent and pose significant public health problems, with children being more affected [6]. In children, these infections have been found to hinder growth, limit physical activity, and impair their cognitive function. Moreover, polyparasitism and the high burden of parasitic infections have a detrimental effect on the overall health of the children [7].

Pre-school-age children, school-age children (SAC), and reproductive-age women are among the group found at high risk for chronic helminthiasis [8]. In developing countries, SAC are more vulnerable to helminthiasis and its associated health consequences due to their playing habits with contaminated soil. Despite the efforts to eliminate these infections among children by 2020, still it remains a significant health problem, particularly in impoverished nations [8].

STHs and leprosy co-morbidity primarily occur in the poorest population living in remote or rural areas where poor hygiene and sanitation practices are common [9]. Compared with other sub-Saharan African countries, Ethiopia faces a considerable challenge in combating NTDs. In Ethiopia, out of 79 million people affected by STHs infections, 25 million are SAC with infection rates ranging from 29.3% to 37.7% [10].

The prevalence of *A. lumbricoides* was higher in Ethiopia's lowlands compared with the highlands, with infection rates of 35% and 29%, respectively. *T. trichiura* had a similar prevalence across all altitudinal zones with an average infection rate of 13%. However, hookworm was more prevalent in the lowlands (24%) followed by temperate zones (15%) and highlands (7%) [7]. The severity of STHs infection is determined by the level of intensity of infection; light and moderate infections often show no symptoms, whereas heavy infections can lead to various negative effects such as malnutrition and delayed physical and cognitive growth [11].

Most of the studies conducted in Ethiopia were school (SAC) and general community-based, and it has been suggested that further study is needed on large-scale communities, especially among those outside of the standard school-based control program, such as marginalized communities living in previous leprosarium villages. Besides, caused by a widespread geographic overlap of different NTDs (leprosy and helminthic co-infection), it is imperative to assess the magnitude of STHs infection in previous leprosarium settlements. To achieve success in the integrated control and elimination of NTDs, it will be crucial to conduct comprehensive mapping, rapid scale-up of interventions, and conducting operational research on the most effective intervention packages. However, in the current study area, there was limited information on the prevalence of STHs infections and its determinants. Therefore, the aim of this study was to compare the prevalence and associated factors of STHs infections among children aged 1-15 years residing in previous leprosarium and non-leprosarium settlements in Eastern Ethiopia.

## Materials and methods

### Study setting

This study was conducted in the Harari Regional State, in Amir Nur District, 01 and 02 Kebeles and Oromia Regional State, and Babile District, Bisidimo and Ifadin Kebeles from November 1, 2023 to February 30, 2024. The Harari Regional State is one of the regions in Ethiopia located 523 km away from Addis Ababa to the east. This region includes nine districts with 19 urban and 17 rural Kebeles. Amir Nur district is one of the urban districts with 5,380 households, 24,215 total population, and 6,041 children aged 1-15 years. Kebele 01 includes 1,398 households and 2,055 children aged 1-15 years old. 02 Kebele includes 1,403 households and 3,986 children aged 1-15 years old. There are 225 leprosy confirmed cases, of whom 135 are male and 90 are female (Amin Nur District Health Office Report, 2023). Ganda Ferro village is one of the villages found in 01 Kebele, and it was established as a leprosarium area that was historically used to isolate and treat individuals suffering from leprosy (Hansen's disease) [12].

Babile district is located 540 km away from Addis Ababa and 23 km from Harar in the eastern direction. Bisidimo Kebele is an area around Bisidimo General Hospital, and it includes 1,496 households, with 7,524 total population, and 3,582 were children aged 1-15 years. Bisidimo Kebele is a historical leprosy settlement area [12]. Patients with leprosy visited Bisidimo General Hospital for treatment and made their residence around this hospital. Ifadin Kebele is a non-leprosarium area around Bisidimo Kebele, and it includes 1,501 households, with 6,627 total population, and 2,178 were children aged 1-15 years (Babile District Health Office Report, 2023). Ethiopia operates a national school-based deworming program aimed at treating SAC for STHs (intestinal worms), targeting a large at-risk population with the goal of eliminating STHs across the country. In the current study area, SAC were treated with mebendazole once a year, and they were earlier treated with this drug 10 months prior to this study.

### Study design and population

A community-based comparative cross-sectional study was conducted. The source population for this study comprised all households having children aged 1-15 years who were living in Amir Nur District; 01 and 02 Kebeles and Babile District; Bisidimo and Ifadin Kebeles. The study population included all children aged 1-15 years who were able to provide a stool sample and their caregivers from selected households who gave full consent to participate. Children who had taken deworming/treatment drugs within the past 2 weeks prior to the data collection and whose caregivers were absent at home or seriously sick and unable to speak during the data collection were excluded from the study.

### Sample size determination and sampling procedure

The sample size was determined by Epi-info version 7.2 software using a double population proportion formula by considering the prevalence of STHs infections (32.3%) from a previous study conducted in Northwest Ethiopia [13] among children aged from 1 to 15 years in non-leprosarium settlements and assuming the prevalence of STHs infection (50%) in a leprosarium settlements, 80% power of the study, 5% margin of error, 95% confidence interval (CI), design effect of 2, population ratio of 1:1, and 10% non-response rate. Thus, the final sample size was 580.

Regarding the sampling procedure; Amir Nur District, 01 Kebele and Babile District, Bisidimo Kebele were selected purposively as previously leprosarium areas and the other two Kebeles were selected randomly as non-leprosarium areas. Households with children aged 1-15 years from each Kebele were selected by systematic sampling technique using a family registration book found at the hands of health extension workers as the sampling frame. To determine the interval of households in the selected Kebeles, the K^th^ value was used (where the K^th^ value was calculated by dividing the total number of households by the calculated sample size [5,798/580 = 10]), and the calculated sample size was proportionally allocated to each Kebele based on the households’ number. The calculated sample size was proportionally allocated to each Kebele based on the households’ number. Finally, the first household was selected by lottery method from one to 10 then continued by selecting every K^th^ value (i.e. 10). In case the selected household had no child/children aged 1-15 years, the next household was used. If there were more than one eligible child at home during data collection, all of them were included. The number of households was proportionally allocated. The total households were almost the same (i.e. 3,105 and 3,095 in household leprosarium and non-leprosarium areas, respectively). Finally, the same number of households (290) from both areas were considered using a systematic sampling technique.

### Data collection methods and laboratory procedures

Data related to both the caregiver's and children's socio-demography and behavioral factors and WASH-related factors of the caregivers were collected by two BSc pediatric nurses using a pre-tested structured questionnaire, which was adapted from previous relevant literature [[14], [15], [16], [17]] through interviewing children's caregivers. A questionnaire was first prepared in English and then translated into local languages (Afan Oromo and Amharic), and again re-translated back into English by different language experts to maintain its consistency. After interview-related questionnaire was completed, all children were provided with marked clean labeled plastic stool cups and requested to bring approximately 5 g of stool specimen [18]. Collected samples were then preserved with 10% formalin at the field setting and an aliquot of each sample was transported to Haramaya University, College of Health and Medical Sciences, Medical Parasitology Teaching Laboratory within the same day using a cold box for examination using formol-ether concentration technique.

For the formol-ether concentration technique, approximately 1 g of feces was added to a clean 15 ml conical test tube containing 7 ml of 10% formalin, and the contents were mixed thoroughly using an applicator stick. The resulting suspension was filtered through a sieve into another conical centrifuge tube. The debris trapped in the sieve was discarded. Then, 3 ml of diethyl ether was added to the formalin solution and placed in the second conical test tube; the contents were centrifuged at medium speed (2500 rpm) for 5 minutes. The supernatant was poured off and the smear was made from the sediment on a clean slide and covered with a clean coverslip. Finally, the smear was examined using 10x and 40x objectives to detect intestinal helminths, particularly STHs ova or larvae and protozoan cysts or oocysts [19]. Cysts of *Giardia lamblia* and *Entamoeba histolytica/Entamoeba dispar* were identified using Lugol's iodine solution. Two senior laboratory professionals performed the laboratory procedures according to the standard operating procedures. In case of any discrepant results during stool examination, another (third) senior laboratory technologist who was blinded to the previous results was invited to confirm the results.

### Data processing and analysis

Collected data were checked for completeness and consistency and entered into Epi-Data version 4.2. Then, exported to SPSS version 26 for analysis. Descriptive statistics, including frequency, mean, or standard deviations, were computed and summarized in texts, tables, and figures. Bivariable and multivariable logistic regression analyses were performed to determine the association between independent variables and STHs infection. Variables with a *P*-value <0.25 in the bivariate analysis were selected as candidates for the final multivariable analysis. A variance inflation factor was used to identify the degree of multicollinearity. Moreover, the model goodness of fit was tested using the Hosmer-Lemeshow statistic; the model was considered a good fit if it was found to be insignificant for the Hosmer-Lemeshow statistic (>0.05). The association between STHs infection and the independent variables was reported as an odds ratio (OR) with its 95% CI and a *P*-value of <0.05 in the final model was considered statistically significant.

### Data quality assurance

The questionnaire was pre-tested on 5% of caregivers at Amir Nur District, Kebele 07 who were not included in the study 1 week before the actual data collection. Based on the results of the pre-test, the required modifications and changes were implemented. Two days of training were given to two supervisors and four data collectors by the research team. The research team was also actively monitoring the fieldwork every day, ensuring that surveys were fully completed and that the data being recorded made sense. All laboratory procedures were performed by following standard operating procedures in the detection and identification of any intestinal parasite stages. In addition, 10% of stool samples were randomly selected and examined by a senior Medical Parasitologist who was blinded to the previous test results.

## Results

### Socio-demographic characteristics

A total of 580 households were included in this study with a response rate of 100%. Among those included households, 290 were from leprosarium settlements and the other 290 were from non-leprosarium settlements. The age range for caregivers was 18 and 60 years with mean ages (±SD) of 31.2 (±7.3) and 32.2 (±7.2) years, in leprosarium and non-leprosarium settlements, respectively. Regarding the educational status of caregivers, 116 (40%) caregivers from leprosarium settlements and 148 (51%) caregivers from non-leprosarium settlements were unable to read and write (Table 1).

**Table 1:** Socio-demographic characteristics and water source, sanitation, and hygiene-related factors of caregivers in previous leprosy and non-leprosy settlements, Eastern Ethiopia, 2024 (n = 580).

Variables		Leprosy settlements (n = 290)		Non-leprosy settlements (n = 290)	
		No	%	No	%
Residence of caregivers	Rural	150	51.7	150	51.7
	Urban	140	48.3	140	48.3
Age of caregivers (in years)	18-24	46	15.9	27	9.3
	25-31	149	51.4	112	38.6
	32-38	52	17.9	103	35.5
	39-45	30	10.3	32	11
	>45	13	4.5	16	5.5
Educational status of caregivers	Unable to read and write	116	40	148	51
	Able to read and write	100	34.5	57	19.7
	Primary school	20	6.9	24	8.3
	Secondary school	24	8.3	6	2
	College and above	30	10.3	55	19
Occupation of caregivers	Farmer	78	26.9	50	17.2
	Merchant	64	22.1	54	18.6
	Employed	60	20.7	64	22.1
	Jobless	30	10.3	14	4.8
Marital status of caregivers	House wife	58	20	108	37.2
	Married	270	93.1	268	92.4
	Separated/divorced	13	4.5	9	3.1
	Widowed	7	2.4	13	4.5
Family monthly income (in Ethiopian birr)	500-3,000	104	35.7	91	31.4
	3,100-5,000	141	48.6	122	42
	5,100-10,000	45	15.5	77	26.6
Family size	≤5	172	59.3	203	70
	>5	118	40.7	87	30
Water source	Pipe	110	37.9	87	30
	Unprotected dug well	112	38.6	101	34.8
	Protected dug well	68	23.5	102	35.2
Latrine availability	Yes	251	86.6	257	88.6
	No	39	13.4	33	11.4
Type of latrine	Open pit	147	50.7	121	41.7
	Ventilated improved pit	39	13.4	31	10.7
	Pit with slab	65	22.4	105	36.2
Waste disposal	Pit	134	46.2	127	43.8
	Open field	156	53.8	163	56.2
Clean utensils for feeding children	With water only	91	31.4	62	21.4
	Water with soap	199	68.6	228	78.6
Hand washing with water and soap before eating food	Yes	230	79.3	249	85.9
	No	60	20.7	41	14.1
Hand washing with water and soap before feeding children	Yes	242	83.4	275	94.8
	No	48	16.6	15	5.2
Hand washing with water and soap after toilet use	Yes	236	81.4	265	91.4
	No	54	18.6	25	8.6
Hand washing with water and soap after helping and cleaning children who had defecated	Yes	273	94.1	269	92.8
	No	17	5.9	21	7.2
Hand washing with water and soap before preparing food	Yes	258	89.9	267	92.1
	No	32	11.1	23	7.9

A total of 838 children (419 from leprosarium settlements and 419 from non-leprosarium settlements) were included in the study. In addition, 221 (52.7%) children from leprosarium settlements and 203 (48.4%) children from non-leprosarium settlements were males. The mean age (±SD) of children in leprosarium and non-leprosarium settlements was 7.21 (±3.77) and 7.58 (±3.63) years, respectively (Table 2).

**Table 2:** Socio-demographic characteristics and childhood-related behavioral factors of children aged 1-15 years in previous leprosy and non-leprosy settlements, Eastern Ethiopia, 2024 (n = 838).

Variables		Leprosy settlements (n = 419)		Non-leprosy settlements (n = 419)	
		No	%	No	%
Residence of children	Rural	214	51.1	206	49.2
	Urban	205	48.9	213	50.8
Sex of children	Male	221	52.7	203	48.4
	Female	198	47.3	216	51.6
Age of children (in years)	1-4	160	32.8	138	32.9
	5-9	176	42	185	44.2
	10-15	83	19.8	96	22.9
Latrine in use practice	Yes	293	69.9	302	72.1
	No	126	31.1	117	27.9
Habit of sharing clothes	Yes	88	68.7	251	59.9
	No	131	31.3	168	40.1
Shoe-wearing habit	Yes	315	75.2	333	79.5
	No	104	24.8	86	20.5
Habit of nail trimming	Yes	286	68.3	307	73.3
	No	133	31.7	112	26.7
History of deworming	Yes	324	77.3	343	81.9
	No	95	22.7	76	18.1
Eating /contact/playing with soil	Yes	159	37.9	113	27
	No	260	62.1	306	73
Eating raw vegetables	Yes	228	54.4	201	48
	No	191	45.6	218	52
Hand washing before eating	Yes	367	87.7	396	94.5
	No	52	12.6	23	5.5
Hand washing after toilet use	Yes	320	76.4	346	82.6
	No	99	23.6	73	17.4

### Water source, sanitation, and hygiene-related factors of the caregivers

The proportion of latrine usage among caregivers in leprosarium and non-leprosarium settlements was 251 (86.6%) and 257 (88.6%), respectively. Regarding solid waste, 134 (46.2%) caregivers from leprosarium settlements and 127 (43.8%) caregivers from non-leprosarium settlements had a pit for solid waste disposal around their homes. Approximately 112 (38.6%) and 101 (34.8%) caregivers from leprosarium and non-leprosarium settlements used unprotected dug wells as a source of drinking water, respectively (Table 1).

### Childhood-related behavioral factors

A total of 159 (37.9%) and 113 (27%) children who were living in leprosarium and non-leprosarium settlements had the habit of eating/contact/playing with soil, respectively. In addition, 315 (75.2%) children from leprosarium settlements and 333 (79.5%) children from non-leprosarium settlements had the habit of shoe-wearing; 286 (68.3%) children from leprosarium settlements and 307 (73.3%) children from non-leprosarium settlements had the habit of nail trimming; 293 (69.9%) children from leprosarium and 302 (72.1%) children from non-leprosarium settlements had latrine in use practice; and 224 (77.3%) children from leprosarium settlements and 343 (81.9%) children from non-leprosarium settlements had history of deworming (Table 2).

### Prevalence of soil-transmitted helminths

**Figure 1:**
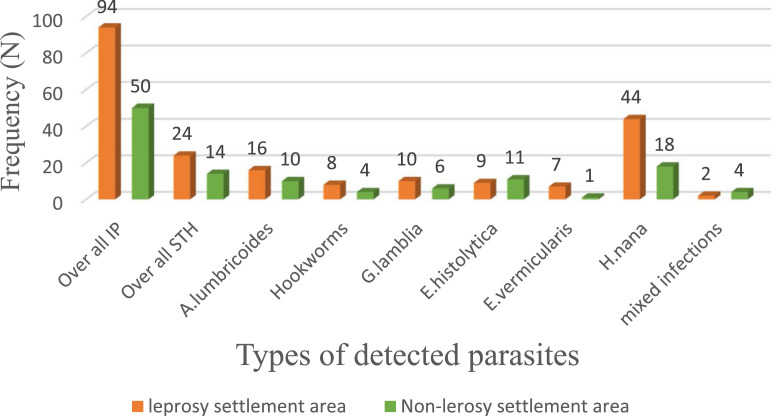
Prevalence of STHs and other intestinal parasites among children aged 1-15 years in previous leprosy and non-leprosy settlements, Eastern Ethiopia, 2024.

The overall prevalence of STHs infection in this study was 38/838 (4.5%) (95% CI 1.31-16.80). Of these, 24 (5.7%) (95% CI 1.2-12.06) were among children from leprosarium settlements and 14 (3.3%) (95% CI 1.32, 12.05) from non-leprosarium settlements. The observed difference was statistically significant (X^2^ = 7.98, *P* = 0.017). The most commonly identified STH was *A. lumbricoides* 26 (3.1%) followed by hookworm 12 (1.4%). Overall, from the intestinal parasitic infections, the most frequently detected helminths were *Hymenolepis nana* 62 (7.4%) followed by *A. lumbricoides* 26 (3.1%). The predominantly detected protozoan parasites were *E. histolytica/E. dispar* 20 (2.4%) and *G. lamblia* 16 (1.9%) (Figure 1).

STHs, soil-transmitted helminths.

### Factors associated with STH infections

#### Among children in previous leprosarium settlements

In bivariate analysis, caregivers-related factors, such as waste disposal practice, latrine availability, hand washing with water and soap, and before feeding children, after helping and cleaning children who had defecated and before preparing food; and children-related factors, such as sex, latrine in use practice, eating/contact/playing with soil, habit of eating raw vegetables, hand washing before eating, and after toilet use, were found to be significantly associated with STHs infection. However, in multivariable analysis, caregivers’ hand washing habit with water and soap after helping and cleaning children who had defecated and children's habit of eating/contact/playing with soil and hand washing before eating and after toilet use remained significantly associated with STHs infection (Table 3).

**Table 3:** Bivariate and multivariable analysis of factors associated with STH infections among children in previous leprosy settlements, Eastern Ethiopia, 2024.

Variables		STHs infection					
		Positive No (%)	Negative No (%)	Crude OR (95% CI)	*P*-value	Adjusted OR (95% CI)	*P*-value
Educational status of caregivers	No formal education	39 (12.5)	273 (87.5)	1.24 (0.61-2.90)	0.27		
	Primary school	3 (10.3)	26 (89.7)	1.68 (1.34-3.72)			
	Second school and	5 (6.4)	73 (93.6)	1			
	above						
Occupation of caregivers	Employed	29 (26.6)	80 (73.4)	1	0.35		
	Unemployed	37 (11.9)	273( 88.1)	2.67 (0.96-4.27)			
Family monthly income	<5,000	55 (20.2)	217 (79.8)	3.13 (0.59-5.63)	0.48		
(In Ethiopian birr)	>5,000	11 (7.5)	136 (92.5)	1			
Family size	<5	27 (10.6)	228 (89.4)	1			
	>5	38 (23.2)	126 (76.8)	0.39 (0.19-0.87)	0.26		
Latrine availability	Yes	45 (12.4)	317 (87.6)	1	0.07	1	0.40
	No	9 (15.9)	48 (84.1)	0.76 (0.41-1.50)		1.90 (0.60-5.91)	
Type of latrine	Open pit	33 (15.6)	179 (84.4)	2.40 (0.86-6.01)	0.52		
	VIP	4 (7.1)	52 (92.9)	0.44 (0.24-2.95)			
	Pit with slab	14 (14.9)	80 (85.1)	1			
Waste disposal	Pit	8 (4.1)	185 (95.9)	1	0.20	1	0.24
	Open field	16 (7.1)	210 (92.9	1.76 (0.74-4.21)		1.69 (0.71-4.06)	
Water source	Pipe	8 (4.6)	167 (95.4)	1	0.65		
	Unprotected dug well	10 (7.3)	127 (92.7)	1.64 (0.63-4.28)			
	Protected dug well	6 (5.6)	101 (94.4)	1.24 (0.42-3.68)			
Children's residence	Rural	12 (5.6)	202 (94.4)	0.96 (0.42-2.8)	0.91		
	Urban	1 2(5.9)	193 (94.1)	1			
Sex of children	Male	8 (3.6)	213 (97.7)	0.43 (0.18-1.02)	0.056	1.72 (0.47-6.35)	0.41
	Female	16 (8.1)	182(91.9)	1		1	
Age of children (in years)	1-5	8 (5.0)	152 (9.0)	1.04 (0.3-3.56)	0.72		
	6-10	12 (6.8)	164 (93.2)	1.45 (0.45-4.62)			
	11-15	4 (4.8)	79 (95.2)	1			
Educational status of children	Under school	8 (7.6)	97 (92.4)	1.20 (0.38-3.83)	0.54		
	Primary and above	11 (4.7)	225 (95.3)	0.71 (0.24-2.12)			
	Not attending school	5 (6.4)	73 (93.6)	1			
Clean utensils for feeding children	With water only	8 (6.3)	119 (93.7)	1.16 (0.48-2.78)	0.74		
	Water with soap	16 (5.5)	276 (94.5)	1			
Hand washing with water and soap before feeding children	Yes	15 (4.7)	303 (95.3)	1	0.12	1	0.71
	No	9 (8.9)	92 (91.1)	1.98 (0.84-4.66)		1.24 (0.4-3.83)	
Hand washing with water and soap after toilet use	Yes	17 (5.4)	297 (94.6)	1	0.63		
	No	7 (6.7)	98 (93.3)	1.25 (0.5-3.09)			
Hand washing with water and soap after helping and cleaning children who had defecated	Yes	15 (4.1)	355 (95.9)	1	0.01	1	0.019
	No	9 (18.4)	40 (81.6)	5.3 (2.2-12.95)		1.26 (1.2-5.8)	
Hand washing with water and soap before preparing food	Yes	14 (4.2)	321 (95.8)	1	0.09	1	0.081
	No	10(11.9)	74 (88.1)	3.1 (1.32-7.25)		1.56 (1.18-6.71)	
Latrine in use	Yes	14 (4.8)	279 (95.2)	1	0.21	1	0.25
	No	10 (7.9)	116 (92.1)	1.72 (0.74-3.98)		0.61 (0.26-1.42)	
Sharing of clothes	Yes	14 (4.9)	274 (95.1)	0.62 (0.27-1.43)	0.26		
	No	10 (7.6)	121 (92.4)	1			
Shoe wearing	Yes	18 (5.7)	297 (94.3)	1	0.98		
	No	6 (5.8)	98 (94.2)	1.01 (0.39-2.62)			
Nail trimming	Yes	15 (5.2)	271 (94.8)	1	0.53		
	No	9 (6.8)	124 (93.2)	1.31 (0.56-3.08)			
History of deworming	Yes	18 (5.6)	306 (94.4)	1	0.78		
	No	6 (6.3)	89 (93.7)	1.15 (0.44-2.97)			
Eating/contact/playing with soil	Yes	16 (10.1)	143 (89.9)	3.52 (1.47-8.44)	0.005	5.98 (1.62-22.1)	0.007
	No	8 (3.1)	252 (96.9)	1		1	
Eating raw vegetables	Yes	9 (3.9)	219 (96.1)	0.48 (0.21-1.13)	0.093	0.32 (0.09-1.13)	0.077
	No	15 (7.9)	176 (92.1)	1		1	
Hand washing before eating	Yes	8 (2.2)	359 (97.8)	1	0.01	1	0.001
	No	16 (30.8)	36 (69.2)	19.9 (7.98-49.8)		3.25 (1.4-11.23)	
Hand washing after toilet use	Yes	9 (2.8)	311(97.2)	1	0.01	1	0.013
	No	15 (15.2)	84 (84.8)	6.2 (2.6-14.59)		1.8 (1.34-7.4)	

CI, confidence interval; OR, odds ratio; STH, soil-transmitted helminths.

The likelihood of STHs infections among children whose caregivers did not wash their hands with water and soap after helping and cleaning children who had defecated was 1.26 times (adjusted OR [AOR]=1.26, 95% CI 1.20-5.80) higher compared with their counterparts. The likelihood of STHs infection among children who had the habit of eating/contact/playing with soil was 5.98 times (AOR = 5.98, 95% CI 1.62-22.10) higher compared with their counterparts. Children who did not wash their hands before eating and after toilet use were 3.25 times (AOR = 3.25, 95% CI 1.40-11.23) and 1.8 times (AOR=1.80, 95% CI 1.34-7.40) more likely to acquire STHs infections compared with their counterparts, respectively (Table 3).

#### Among children in non-leprosarium settlements

In bivariate analysis, caregivers-related factors, such as latrine availability, hand washing with water and soap, and before feeding children, after helping and cleaning children who had defecated and before preparing food; and children-related factors, such as sex, educational status, history of deworming, habit of eating/contact/playing with soil, hand washing before eating and after toilet use, and eating raw vegetables, were significantly associated with STHs infection. However, in multivariable analysis, caregivers’ hand washing habit with water and soap after helping and cleaning children who had defecated and children's hand washing habit before eating remained significantly associated with STHs infection (Table 4).

**Table 4:** Bivariate and multivariable analysis of factors associated with STH infections among children in non-leprosy settlements, Eastern Ethiopia, 2024.

Variables		STHs infection					
		Positive No (%)	Negative No (%)	Crude OR (95%CI)	P-value	Adjusted OR (95%CI)	P-value
Educational status of caregivers	No formal	24(8.1)	272(91.9)	0.68 (0.25-1.94)	0.47		
	Education						
	Primary school	4(11.4)	31(88.6)	3.66 (1.13-9.24)			
	Second school and above	3(3.4)	85(96.6)	1			
Occupation of caregivers	Employed	42(17.3)	201(82.7)	1	0.29		
	Unemployed	23(13.1)	153(86.9)	1.39(0.21-2.43)			
Family monthly income	<5000	53(17.2)	255(82.8)	3.09(0.55-5.59)	0.44		
(In Ethiopian birr)	>5000	7(6.3)	104(93.7)	1			
Family size	<5	36(12.3)	257(87.7)	1			
	>5	14(11.1)	112(88.9)	1.12(0.32-1.25)	0.38		
Latrine availability	Yes	25(6.7)	346(93.3)	1	0.03	1	0.20
	No	5(10.4)	43(89.6)	0.62(0.24-1.36)		1.70(0.40-5.71)	
Type of latrine	Open pit	16(9.1)	159(90.9)	2.16(0.51-4.31)	0.52		
	VIP	2(4.4)	43(95.6)	0.60(0.40-3.15)			
	Pit with slab	11(7.2)	141(92.8)	1			
Waste disposal	Pit	5 (2.8)	171 (97.2)	1	0.63		
	Open field	9 (3.7)	234 (96.3)	1.32 (0.43-3.99)			
Water source	Pipe	6 (3.0)	197 (97.0)	1	0.83		
	Unprotected dug well	5 (4.2)	114 (95.8)	1.44 (0.43-4.82)			
	Protected dug well	3 (3.1)	94 (96.9)	1.05 (0.26-4.28)			
Residence of children	Rural	8 (3.9)	198 (96.1)	1.39 (0.48-4.09)	0.55		
	Urban	6 (2.8)	207 (97.2)	1			
Sex of children	Male	9 (4.4)	194 (95.6)	1.96 (0.65-5.94)	0.24	0.33 (0.05-2.29)	0.26
	Female	5 (2.3)	211 (97.7)	1		1	
Age of children (in years)	1-5	4 (2.9)	134 (97.1)	0.54 (0.14-2.08)	0.52		
	6-10	5 (2.7)	180 (97.3)	0.51 (0.1-1.79)			
	11-15	5 (5.2)	91 (94.8)	1			
Educational status of children	Under school	5 (6.7)	70 (93.3)	1.27 (0.33-4.98)	0.09	1.16 (0.12-11.4)	0.58
	Primary and above	5 (1.9)	264 (98.1)	0.34 (0.09-1.29)		1.7 (0.25-11.36)	
	Not attending	4 (5.3)	71 (94.7)	1		1	
Clean utensils for feeding children	With water only	6 (4.3)	135 (95.7)	1.5 (0.51-4.41)	0.46		
	Water with soap	8 (2.9)	270 (97.1)	1			
Hand washing with water and soap before feeding children	Yes	6 (1.9)	306 (98.1)	1	0.01	1	0.19
	No	8 (7.5)	99 (92.5)	4.12 (1.4-12.17)		1.39 (0.09-1.6)	
Hand washing with water and soap after toilet use	Yes	9 (2.8)	307 (97.2)	1	0.33		
	No	5 (4.9)	98 (95.1)	1.74 (0.57-5.32)			
Hand washing with water and soap after helping and cleaning children who had defecated	Yes	7 (1.8)	376 (98.2)	1	0.02	1	0.001
	No	7 (19.4)	29 (80.6)	12.97 (4.3-39.49)		4.9 (2.2-9.33)	
Hand washing with water and soap before preparing food	Yes	9 (2.8)	318 (97.2)	1	0.22	1	0.26
	No	5 (5.4)	87 (94.6)	2.03 (0.66-6.23)		2.33 (0.53-10.2)	
Latrine in use	Yes	9 (3.0)	293 (97.0)	1	0.51		
	No	5 (4.3)	112 (95.7)	1.45 (0.48-4.436)			
History of deworming	Yes	6 (1.7)	337 (98.3)	1	0.001	1	0.06
	No	8 (10.5)	68 (89.5)	6.61 (2.22-19.6)		0.28 (0.08-0.99)	
Sharing of clothes	Yes	7 (2.8)	244 (97.2)	0.66 (0.23-1.92)	0.45		
	No	7 (4.2)	161 (95.8)	1			
Shoe wearing	Yes	8 (2.4)	325 (97.6)	1	0.44		
	No	6 (7.0)	80 (93.0)	3.05 (1.03-9.03)			
Nail trimming	Yes	9 (2.9)	298 (97.1)	1	0.64		
	No	5 (4.5)	107 (95.5)	1.55 (0.51-4.72)			
Eating/contact/playing with soil	Yes	8 (7.1)	105 (92.9)	3.81 (1.29-11.24)	0.015	2.55 (0.72-8.99)	0.15
	No	6 (2.0)	300 (98.0)	1		1	
Eating raw vegetables	Yes	9 (4.5)	192 (95.5)	2.0 (0.66-6.06)	0.22	3.03 (0.8-11.95)	0.071
	No	5 (2.3)	213 (97.7)	1		1	
Hand washing before eating	Yes	6 (1.5)	390 (98.1	1	0.15	1	0.001
	No	8 (34.8)	15 (65.2)	34.7(10.7-112.5)		18.7 (9.2-38..2)	
Hand washing after toilet	Yes	7 (2.0)	339 (98.0)	1	0.003	1	0.23
	No	7 (9.6)	66 (90.4)	5.14 (1.74-15.13)		1.43 (0.11-1.72)	

CI, confidence interval; OR, odds ratio; STHs, soil-transmitted helminths.

Children whose caregivers did not wash their hands with water and soap after helping and cleaning children who had defecated were 4.9 times (AOR = 4.90, 95% CI 2.21-9.33) more likely to be infected with STHs compared with their counterparts. Children who did not wash their hands before eating were 18.7 times (AOR = 18.70, 95% CI 9.21-38.21) more likely to acquire STHs infection compared with those who had such a habit (Table 4).

## Discussion

The study was conducted to compare the prevalence of STH infections and its associated factors among children aged 1-15 years in the previous leprosarium and non-leprosarium settlements. In this study, the overall prevalence of STHs infections was 4.5% (95% CI 1.31-16.80). The prevalence was 5.7% (95% CI 1.2-12.06) and 3.3% (95% CI 1.32-10.05) among children in previous leprosarium and non-leprosarium areas, respectively. The observed difference was found to be statistically significant (X^2^ = 7.98, *P* = 0.017).

In the current study, the observed overall prevalence of STHs infections among children was low according to the World Health Organization (WHO, 2006) transmission classification; that means the current study area was classified under low STHs transmission (prevalence <20%) [20]. This finding was in line with the previous studies conducted in different parts of Ethiopia: Gurage zone (9.5%) [21], Jimma (7.1%) [21], and Ambo town (12.6%) [22]. However, it was higher than the study conducted in Babile town, Ethiopia (0.47%) [7]. In addition, our study findings were lower compared with previous studies conducted in Jimma (45.6%) [22], Chiro (18.6%) [22], and Sekela primary schools, Ethiopia (25.78 %) [22] and also reported findings from Southeast Nigeria (18.1%) [23], Southern Nairobi (22%) [23], and Western Rwanda (77.7%) [23]. The observed variations might be due to the differences in the parasitologic examination technique that the researchers used, seasonal variation, and the difference in implementing mass deworming activities as an intervention, which may have an important impact on the prevalence of STH infections [23]. The health extension program that has been applied for the last decade in Ethiopia is also supposed to bring changes in the prevalence of STHs infections. Moreover, the differences in the distribution and occurrence of STHs infection in different areas might be due to the differences in environmental factors that favor the transmission cycle of the parasites and egg output [23].

The observed low prevalence of STHs infection in both leprosarium and non-leprosarium settlements might be attributed to the high deworming history of the children before this study was carried out [24]. In leprosy settlements, our finding was comparable with a study conducted in Benin (5.3%) [24]. This might be due to deworming in the previous year were linked to a lower prevalence of STH infections. The prevalence of STHs infections among children in leprosarium settlements was higher than that of children in non-leprosarium settlements. This might be due to leprosarium settlements having an effect in several dimensions on the family's quality of life [23] and also it may lead to low family income [25].

In the present study, *A. lumbricoides* (3.1%) was the most common STH recovered from the children. This finding was consistent with previous study findings reported from South India (3.3%) [5]. The reason behind *A. lumbricoides* predominancy could be associated with the long life of the female worm and her fecundity rate of approximately 134,000-360,000 eggs per day, which persists nearly for 300 days. As a result, massive numbers of eggs are discharged into the human environment daily. Furthermore, the hard nature of these eggs to resist adverse environmental conditions more than other STHs eggs can contribute to sustaining the transmission cycle for a long period of time [17].

In the current study, children's habit of eating/contact/playing with soil was found to be significantly associated with STHs infections in both leprosarium settlements and urban areas. This finding was consistent with a previous study conducted in Bahir Dar Zuria District, Northwest Ethiopia [25]. This could be because STHs infections can be transmitted through ingestion of infective eggs/larvae and also through skin penetration of infective larvae of STHs from contaminated soil with infected human excreta. Thus, having the habit of eating/contact/playing with soil could increase the chance of being infected with STHs, even though eating/contact/playing with soil in itself is not a means of exposure to STHs infections [26].

In our study, caregivers who had no habits of hand washing with water and soap before feeding children and after helping and cleaning children who had defecated and children who had no habit of hand washing before eating and after toilet use were significantly associated with STHs infections. These findings were in line with previous results reported from Debre Tabor, Western Ethiopia [27], and North Sumatera, Indonesia [15]. The observed association in our study might be related to the poor access to improved WASH practice that aggravates the occurrence of STHs infections in children [28].

In the present study, variables like having the habit of eating raw vegetables and having no fingernail trimming and shoe-wearing habits were not significantly associated with STHs infections. In contrast to our findings, previous studies conducted in the Tachgayint District of Northcentral Ethiopia [18], Bahir Dar Zuria District [26], and Uganda [16] showed a significant association between those factors and STHs infections among children. The possible justification for the absence of an association in our study could be due to the low prevalence of STHs infections observed [13].

The strength of this study is that it was conducted in a wider population (children) living in previous leprosarium and non-leprosarium settlements. In addition, because it was a community-based study with systematic random sampling of the children, it would be possible to generalize the study's findings to all children living in the study areas. However, our study might have some limitations. The cross-sectional nature of the study design does not confirm the definitive cause-and-effect relationship. Even though measuring the intensity of STHs infections, especially in low prevalence settings is important, we did not perform the Kato-Katz technique due to budget constraints. Moreover, this study also did not consider other diagnostic techniques that are best to estimate the prevalence of STHs and identify different species of hookworms due to the same reason related to budget. Because the study was community-based, the wet mount technique was not performed to detect *Strongyloides stercoralis* larvae; this might have caused to miss this species, which in turn resulted in the low prevalence of STHs infections observed in our study.

## Conclusion

In this study, the prevalence of STHs infections among children aged 1-15 years in the study areas was low based on the World Health Organization (2006) transmission classification. However, the prevalence of STHs infections was found to be high among children living in previous leprosarium compared with non-leprosarium settlements. Having no habit of hand washing with water and soap after helping and cleaning children who had defecated, having the habit of eating/contact/playing with soil and having no habit of hand washing before eating and after toilet use were significantly associated with STHs infections. Therefore, targeted mass deworming and health information dissemination should be given to caregivers and their children on the importance of proper hygiene and sanitation to alleviate the problem. Our study also highlights the need for further large-scale study that mainly emphasizes the intervention strategies for STHs infections among marginalized communities including leprosarium areas in Ethiopia.

## Declarations of competing interest

The authors have no competing interests to declare.
